# A Novel Tensor Ring Sparsity Measurement for Image Completion

**DOI:** 10.3390/e26020105

**Published:** 2024-01-24

**Authors:** Junhua Zeng, Yuning Qiu, Yumeng Ma, Andong Wang, Qibin Zhao

**Affiliations:** 1 School of Automation, Guangdong University of Technology, Guangzhou 510006, China; jh.zenggdut@gmail.com (J.Z.); yuning.qiu.gd@gmail.com (Y.Q.); yumengmaymm@gmail.com (Y.M.); 2 RIKEN Center for Advanced Intelligence Project (AIP), Tokyo 103-0027, Japan; andong.wang@riken.jp

**Keywords:** sparse modeling, tensor sparsity measurement, TR decomposition, tensor completion

## Abstract

As a promising data analysis technique, sparse modeling has gained widespread traction in the field of image processing, particularly for image recovery. The matrix rank, served as a measure of data sparsity, quantifies the sparsity within the Kronecker basis representation of a given piece of data in the matrix format. Nevertheless, in practical scenarios, much of the data are intrinsically multi-dimensional, and thus, using a matrix format for data representation will inevitably yield sub-optimal outcomes. Tensor decomposition (TD), as a high-order generalization of matrix decomposition, has been widely used to analyze multi-dimensional data. In a direct generalization to the matrix rank, low-rank tensor modeling has been developed for multi-dimensional data analysis and achieved great success. Despite its efficacy, the connection between TD rank and the sparsity of the tensor data is not direct. In this work, we introduce a novel tensor ring sparsity measurement (TRSM) for measuring the sparsity of the tensor. This metric relies on the tensor ring (TR) Kronecker basis representation of the tensor, providing a unified interpretation akin to matrix sparsity measurements, wherein the Kronecker basis serves as the foundational representation component. Moreover, TRSM can be efficiently computed by the product of the ranks of the mode-2 unfolded TR-cores. To enhance the practical performance of TRSM, the folded-concave penalty of the minimax concave penalty is introduced as a nonconvex relaxation. Lastly, we extend the TRSM to the tensor completion problem and use the alternating direction method of the multipliers scheme to solve it. Experiments on image and video data completion demonstrate the effectiveness of the proposed method.

## 1. Introduction

Image completion is a well-known problem in the field of image processing, which aims to recover the missing entries of partially observed image data [1,2]. Image completion methods have extensive applications in practical scenarios, including hyperspectral image recovery [3] and video inpainting [4]. These techniques have also exhibited successful implementations across various domains within the natural sciences. For example, in [5,6], they used image completion methods to achieve reliable, high-quality seismic data, which is crucial before the subsequent processing steps. Moreover, in [7], they used the image completion method to acquire dense earthquake data, which largely promotes the understanding of the structure and dynamics of Earth.

Sparsity is an important property in data representation and is extremely important for data analysis. Examples include Anisotropic interaction rules recognition in biological groups [8], face modeling [9], image compressive sensing [10,11], MRI compressive sensing [12,13,14], signal restoration [15,16], etc. For image completion, it is important to capture the inherent sparsity of the image data for the successful recovery of the missing entries. A common method of measuring the sparsity of the data uses the rank of the matrix, and it essentially measures the numbers of second-order Kronecker bases: $u_{i}v_{i}^{T}$ ($u_{i}$ and $v_{i}$ are vectors), i=1,…,R, used for matrix representation, where *i* is an index referring to the *i*-th Kronecker basis, and *R* is the matrix rank. The image completion task, thus, can be formulated as an optimization problem with the objective of minimizing the rank of the candidate matrices. However, the optimization problem involving rank minimization is NP-hard due to its discrete nature, and the trace norm is widely used as the convex surrogate [17,18]. Successful application of trace norm minimization for image completion can be found in [19]. Furthermore, it has been proved theoretically that the rank minimization problem can be solved by the trace norm minimization problem under some reasonable conditions [20].

However, in practice, images are usually generated from the interaction of multiple factors. While a matrix is well-suited for representing data arising from the interaction of two factors, employing a matrix rank for image completion in such cases needs to transform the data into a matrix format. This transformation may break the multi-mode correlation of the data and result in sub-optimal performance [21]. As a high-order generalization of a matrix, a tensor provides a natural way to represent these multi-way data and has been widely used for data representation in many areas such as electrodynamics [22,23], signal processing [24,25,26], and computer vision [27,28,29]. One of the most important tensor analysis techniques is tensor decomposition, which provides a compact representation of the tensor. The most classical tensor decomposition method is called CANDECOMP/PARAFAC or canonical polyadic (CP) decomposition, which was proposed by Carroll et al. [30]. CP decomposition decomposes a tensor into a sum of the rank-one tensor $\sum_{r=1}^{R}a_{r}^{\left(1\right)}\circ \cdots \circ a_{r}^{\left(d\right)}$, where $a_{r}^{\left(k\right)},k=1,\ldots ,d$ are vectors, *d* is the order of the tensor, R is the CP rank, and ∘ is the outer product operation. It can be observed that the CP rank serves as a natural generalization for the sparsity measurement of the tensor since matrix decomposition and the matrix rank are the special cases of CP decomposition when d=2. However, unlike its lower-order counterpart, the computation of the CP rank is an NP-hard problem [31], and it is also difficult to establish a solvable relaxation form [32].

Besides CP decomposition, many studies focus on the Tucker decomposition proposed in [33], which decomposes a tensor into a core tensor multiplied by factor matrices along each mode. Image completion algorithms based on low-rank Tucker decomposition can be found in [34,35]. Recently, tensor network decomposition, initially derived from the physics community for quantum many-body simulations, has been brought to the signal processing community and is emerging as a powerful tool for the analysis of tensor data [36,37]. Among them, the tensor train (TT) decomposition [38], the quantized tensor train (QTT) decomposition [39], and the tensor ring (TR) decomposition [40] have achieved the most research interests. Many low-rank TT and TR completion algorithms have been developed [41,42,43], and the experiment results demonstrate the superiority of the tensor network methods compared with Tucker decomposition methods in image completion, which is mainly due to the powerful representation ability of the tensor network decomposition.

**Figure 1:**
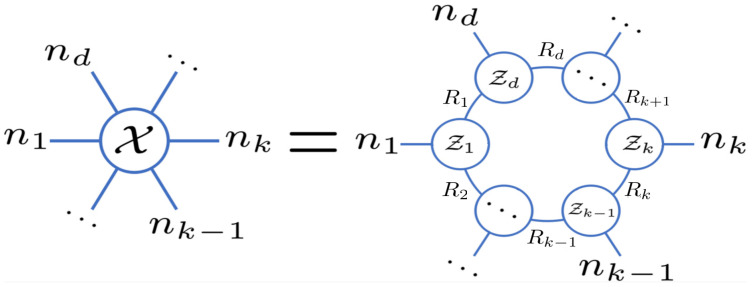
Illustrations of the TR decomposition.

**Figure 2:**
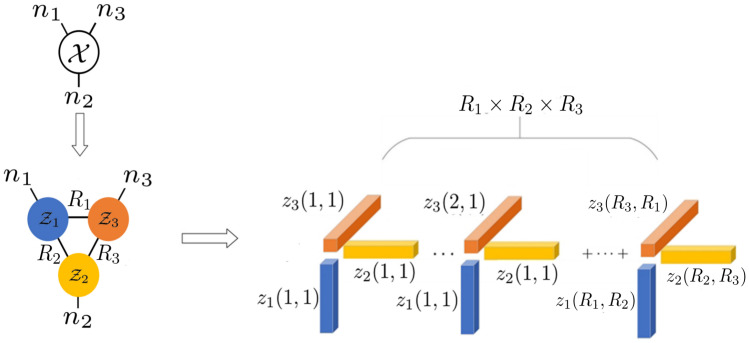
Illustrations of TR decomposition and its Kronecker bases representation.

While Tucker, TT, and TR decomposition ranks are commonly employed in tensor data analysis, they lack a similar interpretation of tensor sparsity as seen in CP rank. To bridge this gap, several studies measure the sparsity of the tensor using the Kronecker bases constructed by the factor matrices of the Tucker decomposition [44,45,46,47]. Different from the previous studies, and inspired by the strong expressiveness of the tensor network, in this work, we focus on TR decomposition, as shown in Figure 1, and the main contributions of the paper are the following:

- We define a novel tensor sparsity measurement, termed tensor ring sparsity measurement (TRSM), which can be efficiently computed by the continuous product of ranks of the TR cores. Specifically, it measures the sparsity of tensors as the numbers of the Kronecker bases constructed by the TR cores for tensor representation. The graphical demonstration of the Kronecker bases representation of a tensor based on TR decomposition is shown in Figure 2.
- To improve the practicality of TRSM, the minimax concave penalty (MCP) folded-concave penalty is introduced as a nonconvex relaxation and then applied to the tensor completion problem, which has previously been applied in computer vision and pattern recognition. As a result, we formulate a new tensor completion model called tensor ring sparsity measurement tensor completion (TRSM-TC). An efficient algorithm based on the alternating direction method of multipliers (ADMM) is developed to optimize the proposed model. Experiments show that TRSM-TC achieves better performance than other algorithms in recovering a high missing rate hyperspectral images and video.

The remainder of this paper is organized as follows. Section 2 introduces the preliminaries of tensor algebra and TR decomposition. Section 3 presents the proposed TRSM. The TRSM-TC problem and its optimization are formulated in Section 4. The complexity analysis is presented in Section 5. Section 6 demonstrates the experimental results and is followed by the conclusions in Section 7.

## 2. Preliminaries

### 2.1. Tensor Algebra

In this paper, some notations and preliminaries of tensors [48] are adopted. Scalars, vectors, and matrices are denoted by lowercase letters (e.g., x∈R), boldface lowercase letters (e.g., $x\in R^{n_{1}}$), and capital letters (e.g., $X\in R^{n_{1}\times n_{2}}$), respectively. A tensor is a multidimensional array and is denoted by calligraphic letters, (e.g., $X\in R^{n_{1}\times \cdots \times n_{d}}$), where $n_{i}$ is the size of the corresponding mode. $X(i_{1},i_{2},\ldots ,i_{n})$ or $x_{i_{1}i_{2}\cdots i_{n}}$ denote an element of tensor $X\in R^{n_{1}\times \cdots \times n_{d}}$ in position $\left(i_{1},i_{2},\ldots ,i_{n}\right)$. A mode-k fiber of tensor $X\in R^{n_{1}\times \cdots \times n_{d}}$ is a vector denoted as $x_{i_{1}\cdots i_{k}-1:i_{k}+1\cdots i_{d}}$, where a colon is used to indicate all elements of a mode. A tensor sequence of $\left\{X^{\left(1\right)},\ldots ,X^{\left(d\right)}\right\}$ can be denoted as ${\left\{X^{\left(k\right)}\right\}}_{k=1}^{d}$. Where appropriate, a tensor sequence can also be written as [X]. The matrix sequences and vector sequences can be defined similarly. The inner product of two tensors X, Y with the same size is defined as $\langle X,Y\rangle =\sum_{i_{1}}\sum_{i_{2}}\cdots \sum_{i_{d}}x_{i_{1}i_{2}\cdots i_{d}}y_{i_{1}i_{2}\cdots i_{d}}$. Furthermore, the Frobenius norm of X is defined by ${∥X∥}_{F}=\sqrt{\langle X,X\rangle }$.

Two types of tensor unfolding expressions are defined in this article. The mode-k unfolding of tensor $X\in R^{n_{1}\times \cdots \times n_{d}}$ converts a tensor to a matrix, which is denoted as $X_{\left(k\right)}\in R^{n_{k}\times n_{1}\cdots n_{k-1}n_{k+1}\cdots n_{d}}$, and using the multi-indices defined in [36], its elements are defined by $X_{\left(k\right)}\left(i_{k},\bar{i_{1}\cdots i_{k-1}i_{k+1}\cdots i_{d}}\right)=X\left(i_{1},i_{2},\ldots ,i_{d}\right)$ Another mode-k unfolding of tensor $X\in R^{n_{1}\times \cdots \times n_{d}}$ is denoted as $X_{\left[k\right]}\in R^{n_{k}\times n_{k+1}\cdots n_{d}n_{1}\cdots n_{k-1}}$, and its elements are defined by $X_{\left[k\right]}\left(i_{k},\bar{i_{k+1}\cdots i_{d}i_{1}\cdots i_{k-1}}\right)=X\left(i_{1},i_{2},\ldots ,i_{d}\right)$. Furthermore, the inverse operation of tensor unfolding is matrix folding, which transforms matrices to higher-order tensors as an inverse operation of the corresponding tensor unfolding. In this paper, we only define the folding operation ${\mathrm{fold}}_{k}\left(\cdot \right)$ for the first type of mode-k unfolding as ${\mathrm{fold}}_{k}\left(X_{\left(k\right)}\right)=X$.

### 2.2. Tensor Ring Decomposition

TR decomposition is proposed to represent a high-order tensor by a sequence of three-order tensors that are multiplied together circularly. Specifically, given a *d*-order tensor $X\in R^{n_{1}\times \cdots \times n_{d}}$, TR decomposition decomposes it into a sequence of latent tensors $Z_{k}\in R^{R_{k}\times n_{k}\times R_{k+1}},k=1,\ldots ,d$, which can be expressed in an element-wise form given by

(1) $$X\left(i_{1},i_{2},\ldots ,i_{d}\right)=\mathrm{Tr}\left\{Z_{1}\left(i_{1}\right)Z_{2}\left(i_{2}\right)\cdots Z_{d}\left(i_{d}\right)\right\},$$

$Z_{k}\left(i_{k}\right)$ is the $i_{k}$th lateral slice matrix of the latent tensor $Z_{k}$, which is of size $R_{k}\times R_{k+1}$. The last latent tensor $Z_{d}$ is of size $R_{d}\times n_{d}\times R_{1}$, where $R_{d+1}=R_{1}$. $Z_{k}$ are called TR cores. The collection of $R_{k},\;k=1,2,\ldots ,d,$ is defined as TR ranks. The illustration of TR decomposition is shown in Figure 1.

## 3. Tensor Ring Sparsity Measurement

By expressing the TR decomposition of Equation (1) in the tensor form, we obtain the following expression

(2) $$X=\sum_{r_{1},\ldots ,r_{d}}^{R_{1},\ldots ,R_{d}}z_{1}\left(r_{1},r_{2}\right)\circ z_{2}\left(r_{2},r_{3}\right)\circ \ldots \ldots \circ z_{d}\left(r_{d},r_{1}\right),$$

where $z_{k}\left(r_{k},r_{k+1}\right)$ is the mode-2 fiber of the TR core $Z_{k}$. As we can see, based on TR decomposition, a tensor X can be represented by $\prod_{i=1}^{d}R_{i}$ Kronecker bases constructed by the TR-cores. In Figure 2, we provide a graphical demonstration of this representation over a three-order tensor. In consequence, when considering tensors represented through TR decomposition, the total numbers of the Kronecker bases required in Equation (2) naturally provide a way to measure the sparsity of the tensor. In this work, we utilize the following formulation to measure the Kronecker bases sparsity of a tensor X defined upon the TR decomposition

(3) $$K\left(X\right)=\prod_{i=1}^{d}\mathrm{rank}\left(Z_{i(2)}\right).$$

The following theorem explains the relation between K(X) and the TR Kronecker bases sparsity of the tensor

**Theorem** **1.** *Given a d-th order tensor $X\in R^{n_{1}\times \cdots \times n_{d}}$, which can be represented by Equation (1), the following inequality holds*

(4) $$\sqrt{\prod_{i=1}^{d}\mathrm{rank}\left(Z_{i(2)}\right)}\leq \prod_{i=1}^{d}R_{i}.$$

**Proof.**  For the mode-2 unfolding TR cores $Z_{i(2)}\in R^{n_{i}\times R_{i}R_{i+1}}$, according to the property of matrix rank, the following inequality holds

(5) $$\mathrm{rank}\left(Z_{i(2)}\right)\leq \min\left\{n_{i},R_{i}R_{i+1}\right\}\leq R_{i}R_{i+1}.$$

The proof is completed by

(6) $$\sqrt{\prod_{i=1}^{d}\mathrm{rank}\left(Z_{i(2)}\right)}\leq \sqrt{\prod_{i=1}^{d}R_{i}^{2}}=\prod_{i=1}^{d}R_{i}.$$

 □

We can conclude that $\sqrt{\prod_{i=1}^{d}\mathrm{rank}\left(Z_{i(2)}\right)}\;$ serves as the lower bound of the TR Kronecker bases sparsity of the tensor. However, due to the lack of a straightforward optimization method for handling the matrix optimization problem involving a square root. We drop the square root and relax it to the form of K(X). Moreover, the optimization of $\mathrm{rank}(Z_{i(2)})$ will lead to combinatorial optimization when applied. Instead of using the trace norm as the convex surrogate to ensure the practical performance of the proposed method, in this work, we use the nonconvex relaxations over the rank of the matrix. Specifically, the MCP folded-concave penalty [49] is chosen here due to its nearly unbiased statistical properties in variable selection. As a result, we can obtain the following relaxation over K(X)

(7) $$K_{\mathrm{MCP}}\left(X\right)=\prod_{i=1}^{d}{\mathrm{rank}}_{\mathrm{MCP}}\left(Z_{i(2)}\right).$$

Here, ${\mathrm{rank}}_{\mathrm{MCP}}\left(\cdot \right)$ denotes the MCP folded-concave penalties of the matrix, which is defined as

(8) $${\mathrm{rank}}_{\mathrm{MCP}}\left(X\right)=\sum_{i}\psi_{\mathrm{mcp}}\left(\sigma_{i}\left(X\right)\right),$$

where $\sigma_{i}\left(X\right)$ denotes the *i*-th singular value of matrix X and

(9) $$\psi_{\mathrm{mcp}}\left(t\right)=\left\{\begin{array}{cc} \lambda |t|-\frac{t^{2}}{2a}, & \;\mathrm{if}\;|t|<a\lambda \\ a\lambda^{2}/2, & \;\mathrm{if}\;|t|\geq a\lambda . \end{array}\right.$$

The function $\psi_{\mathrm{mcp}}\left(\cdot \right)$ is the MCP folded-concave penalty, which can be seen as a continuous interpolation between the $\ell_{1}$ penalty when a=∞, and the $\ell_{0}$ penalty when a→0+. Therefore, applying the MCP penalty to the singular values will result in a tighter surrogate of the matrix rank compared to the nuclear norm. Here, λ and *a* are two hyperparameters that play different roles in determining the shape of the $\psi_{\mathrm{mcp}}\left(\cdot \right)$. *a* mainly controls the concavity of the function, while λ controls the penalty level [49].

## 4. Tensor Ring Sparsity Measurement-Based Tensor Completion

The core problem of the missing value estimation lies in how to build up the relationship between the known elements and the unknown ones. In this paper, we incorporate the proposed TRSM in the tensor completion problem as the optimization objective and formulate the TRSM-TC model as

(10) $$\begin{array}{c} \min_{[Z],X}\prod_{i=1}^{d}{\mathrm{rank}}_{\mathrm{MCP}}\left(Z_{i(2)}\right)+\frac{\lambda }{2}{∥X-\Psi \left(\left[Z\right]\right)∥}_{F}^{2}\\ \;\;\;s.t.P_{\Omega }\left(X\right)=P_{\Omega }\left(T\right), \end{array}$$

where $P_{\Omega }\left(T\right)$ denotes all the observed entries with respect to the set of indices of observed entries represented by Ω, T denotes the observed tensor, and Ψ([Z]) denotes the TR format tensor generated from [Z]. Every element of Ψ([Z]) is calculated by Equation (1).

Due to the complex folded-concave penalties, in order to solve the model, a slightly modified ADMM inspired by [46] is needed. In the modified ADMM, the solution process over Equation (10) is divided into two stages. In the first stage, we use ADMM to solve the trace norm version of Equation (10), demonstrated as follows

(11) $$\begin{array}{c} \min_{[Z],[M],X}\prod_{i=1}^{d}∥M_{i(2)}{∥}_{*}+\frac{\lambda }{2}{∥X-\Psi \left(\left[Z\right]\right)∥}_{F}^{2}\\ \;\;\;\;\;s.t.P_{\Omega }\left(X\right)=P_{\Omega }\left(T\right),M_{i(2)}=Z_{i(2)},i=1,2,\ldots ,d. \end{array}$$

Here, we introduce [M] as auxiliary variables over the TR-cores, and the updated values of $M_{i(2)}$, for i=1,…,d will be used in the subsequent optimization. It is worth mentioning that the first stage of ADMM is only running for one iteration. In the second stage, we instead solve the following problem

(12) $$\begin{array}{c} \min_{[Z],[M],X}\prod_{i=1}^{d}\hat{{\mathrm{rank}}_{\mathrm{MCP}}}\left(M_{i(2)}|M_{i(2)}^{0}\right)+\frac{\lambda }{2}{∥X-\Psi \left(\left[Z\right]\right)∥}_{F}^{2}\\ \;\;\;\;\;s.t.P_{\Omega }\left(X\right)=P_{\Omega }\left(T\right),M_{i(2)}=Z_{i(2)},i=1,2,\ldots ,d. \end{array}$$

Here, $\hat{{\mathrm{rank}}_{\mathrm{MCP}}}\left(X|Y\right)=\sum_{i}{\hat{\psi }}_{\mathrm{MCP}}\left(\sigma_{i}\left(X\right)|\sigma_{i}\left(Y\right)\right)$, and ${\hat{\psi }}_{\mathrm{MCP}}\left(\sigma_{i}\left(X\right)|\sigma_{i}\left(Y\right)\right)=\psi_{\mathrm{MCP}}\left(\sigma_{i}\left(Y\right)\right)+\psi_{\mathrm{MCP}}^{\prime }\left(\sigma_{i}\left(Y\right)\right)\left(\sigma_{i}\left(X\right)-\sigma_{i}\left(Y\right)\right)$, $\psi_{\mathrm{MCP}}^{\prime }\left(\cdot \right)$ is the derivative of $\psi_{\mathrm{MCP}}\left(\cdot \right)$. For $M_{i}^{0},i=1,\ldots ,d$, they are the fixed parameters of the optimization problem. Moreover, the values of $M_{i}^{0}$ are assigned from the $M_{i}$ obtained in the first stage element-wisely, for i=1,…,d. For Equation (12), the augmented Lagrangian function is

(13) $$\begin{array}{cc} L([Z],X,[M],[Y])= & \prod_{i=1}^{d}\hat{{\mathrm{rank}}_{\mathrm{MCP}}}\left(M_{i(2)}|M_{i(2)}^{0}\right)+(\sum_{k=1}^{d}\frac{\mu }{2}∥M_{k}-Z_{k}+\frac{1}{\mu }Y_{k}{∥}_{F}^{2})\\ & +\frac{\lambda }{2}{∥X-\Psi \left(\left[Z\right]\right)∥}_{F}^{2}\\ & s.t.P_{\Omega }\left(X\right)=P_{\Omega }\left(T\right), \end{array}$$

where [Y] are the Lagrangian multipliers, and μ>0 is a penalty parameter. For k=1,…,d, $Z_{k}$, $M_{k}$, $Y_{k}$ are all independent, and we update them using the following updating schemes.

**Update of** $Z_{k}$. For k=1,…,d, the augmented Lagrangian function with respect to $Z_{k}$ can be simplified as

(14) $$\begin{array}{cc} & L\left(Z_{k}\right)=\frac{\mu }{2}∥M_{k}-Z_{k}+\frac{1}{\mu }Y_{k}{∥}_{F}^{2}+\frac{\lambda }{2}{∥X-\Psi \left(\left[Z\right]\right)∥}_{F}^{2}+C_{Z_{k}}, \end{array}$$

where the constant $C_{Z_{k}}$ consists of other parts of the Lagrangian function, which are irrelevant to updating $Z_{k}$. This is a least squares problem, and the best result is obtained when the derivative of Equation (14) equals zero, so for k=1,…,d, $Z_{k}$ can be updated by

(15) $$Z_{k}={\mathrm{fold}}_{2}\left(\left(\mu M_{k(2)}+Y_{k(2)}+\lambda X_{\left[k\right]}\left(Z_{\left[2\right]}^{\neq k}\right)\right){\left(\mu I+\lambda {\left(Z_{\left[2\right]}^{\neq k}\right)}^{T}\left(Z_{\left[2\right]}^{\neq k}\right)\right)}^{-1}\right),$$

where $I\in R^{R_{k}^{2}\times R_{k}^{2}}$ denotes the identity matrix, and $Z_{\left[2\right]}^{\neq k}$ is the subchain of TR decomposition with respect to mode-k, where the definition can be found in [40].

**Update of** $M_{i}$. For i=1,…,d, the augmented Lagrangian functions with respect to $M_{i}$ is expressed

(16) $$\begin{array}{c} L\left(M_{i}\right)=a_{i}\hat{{\mathrm{rank}}_{\mathrm{MCP}}}\left(M_{i(2)}|M_{i(2)}^{0}\right)+<Y_{i},M_{i}-Z_{i}>+\frac{\mu }{2}{∥M_{i}-Z_{i}∥}_{F}^{2}+C_{M_{i}}, \end{array}$$

where $a_{i}=\prod_{k=1,k\neq i}^{d}\hat{{\mathrm{rank}}_{\mathrm{MCP}}}\left(M_{k(2)}|M_{k(2)}^{0}\right).$ Here, we introduce $a_{i}$ as an auxiliary variable for saving space to represent the constant factor that is not related to the optimization variable $M_{i}$. The above formulation has a closed-form solution, which is given by

(17) $$M_{i}={\mathrm{fold}}_{2}\left({\hat{D}}_{\frac{a_{i}}{\mu },v_{i}}\left(Z_{i(2)}-\frac{1}{\mu }Y_{i(2)}\right)\right),$$

where $v_{i}=(\psi_{\mathrm{MCP}}^{\prime }\left(\sigma_{1}\left(M_{i(2)}^{0}\right)\right),\ldots ,\psi_{\mathrm{MCP}}^{\prime }{\left(\sigma_{r}\left(M_{i(2)}^{0}\right)\right)}^{T}$ is a collection of the singular values of $M_{i(2)}^{0}$ and ${\hat{D}}_{r,w}\left(X\right)=U\Sigma_{r,w}V^{T}$ is the generalized shrinkage operator over *X* where $\Sigma_{r,w}=\mathrm{diag}\left(\max\left(\sigma_{i}-rw_{i},0\right)\right)$ ($\sigma_{i}$ is the *i*-th singular value of *X*, and $w_{i}$ is the *i*-th element of w).

**Update of** X. The augmented Lagrangian functions with respect to X are given by

(18) $$\begin{array}{cc} L(X)= & \frac{\lambda }{2}{∥X-\Psi \left(\left[Z\right]\right)∥}_{F}^{2}+C_{X}\\ & s.t.P_{\Omega }\left(X\right)=P_{\Omega }\left(T\right). \end{array}$$

The recovery tensor X is updated by inputting the observed values in the corresponding entries and by approximating the missing entries by the updated TR factors [Z] for every iteration.

(19) $$X=P_{\Omega }\left(T\right)+P_{\bar{\Omega }}\left(\Psi \left(\left[Z\right]\right)\right),$$

where $\bar{\Omega }$ is the set of indices of missing entries, which is a complement to Ω.

**Update of** $Y_{k}$. For k=1,…,d and the Lagrangian multiplier, $Y_{k}$ is updated as

(20) $$Y_{k}=Y_{k}+\mu \left(M_{k}-Z_{k}\right).$$

The penalty term of the Lagrangian functions *L* is restricted by μ, and it is updated for every iteration by $\mu =\min(\rho \mu ,\mu_{\max})$, where 1<ρ<1.5 is a tuning hyperparameter. Moreover, at the end of each iteration, the values of $M_{i(2)}$ will be assigned to $M_{i(2)}^{0}$ element-wisely as an update of $M_{i(2)}^{0}$, for i=1,…,d.

**Figure 3:**
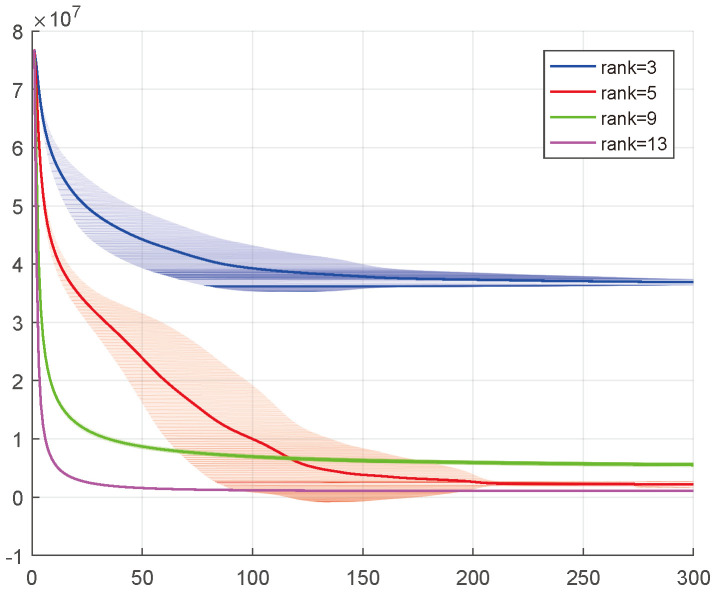
Illustration of convergence property for TRSM-TC under different choices of TR-ranks. A synthetic tensor with TR structure (size (7×8×7×8×7) with TR-rank [5,5,5,5,5], missing rate 0.8) is tested. The experiment records the change in the objective function values along the number of iterations. Each independent experiment is conducted 100 times, the average results are shown in the graphs, and the convergence curve is presented.

The ADMM-based solving scheme is updated iteratively based on the above updating scheme. Two optimization stopping conditions are set: (i) maximum number of iterations maxiter and (ii) the difference between two iterations (i.e., ${∥X-X_{\mathrm{last}}∥}_{F}/{∥X∥}_{F}$), which is thresholded by the tolerance tol. The implementation process of TRSM-TC is summarized in Algorithm 1. The convergent property of the proposed algorithm is demonstrated in Figure 3.
**Algorithm 1** Tensor Ring Sparsity Measurement Tensor Completion**Input:** $P_{\Omega }\left(T\right)$, ${\left\{Z_{k}\right\}}_{k=1}^{d}$: initial TR-cores, ${\left\{M_{i}\right\}}_{i=1}^{d}$: initial auxiliary variables, ${\left\{Y_{i}\right\}}_{i=1}^{d}$: initial Lagrangian multiplier, initial X, λ, μ, $\mu_{max}$, ρ, tol, maxiter **Output:** completed tensor X and TR-cores ${\left\{Z_{k}\right\}}_{k=1}^{d}$
 1:$M_{i}^{0}=M_{i}$ for i=1,…,d, where $M_{i},\;i=1,\ldots ,d$ are obtained from one iteration ADMM update of Equation (11); 2:**for** i=1 to maxiter **do** 3:   **if** i>1 **then** 4:       $M_{i}^{0}=M_{i}$ for i=1,…,d; 5:   **end if** 6:   Update ${\left\{Z_{k}\right\}}_{k=1}^{d}$ by Equation (15); 7:   Update ${\left\{M_{i}\right\}}_{i=1}^{d}$ by Equation (17); 8:   $X_{last}=X$; 9:   Update X by Equation (19);10:   Update ${\left\{Y_{i}\right\}}_{i=1}^{d}$ by Equation (20);11:   $\mu =max\left(\rho \mu ,\mu_{max}\right)$;12:   **if** ${∥X-X_{last}∥}_{F}/{∥X∥}_{F}<tol$ **then**13:       break;14:   **end if**15:**end for**


## 5. Computational Complexity

We analyze the computational complexity of the proposed TRSM-TC as follows. For a tensor $X\in R^{n_{1}\times \cdots \times n_{d}}$, the TR-ranks are set as $R_{1}=R_{2}=\cdots =R_{d}=j$, and the main computational complexity in updating auxiliary variables [M] comes from the SVD operation, which is $𝒪(\sum_{k=1}^{d}2\left(d-1\right)n_{k}^{2}j^{2})$. The computational complexities of calculating $Z_{\left[2\right]}^{\neq k}$ and updating [Z] are $𝒪(dj^{3}\prod_{i=1,i\neq k}^{d}n_{i})$ and $𝒪(dj^{2}\prod_{i=1}^{d}n_{i}+dj^{6})$, respectively. If we assume $n_{1}=n_{2}=\cdots =n_{d}=n$, then the overall complexity of the proposed algorithm can be written as $𝒪(dj^{2}n^{d}+dj^{6})$.

## 6. Experimental Results

In this section, tensor completion algorithms, including Tmac [50], FBCP [51], CP-WOPT [52], HaLRTC [53], TRLRF [42], TR-WOPT [54], are chosen as the baseline methods, and we evaluate the effectiveness of the proposed TRSM-TC method on color images, multispectral images, and video data completion.

### 6.1. Color Images Completion

**Figure 4:**
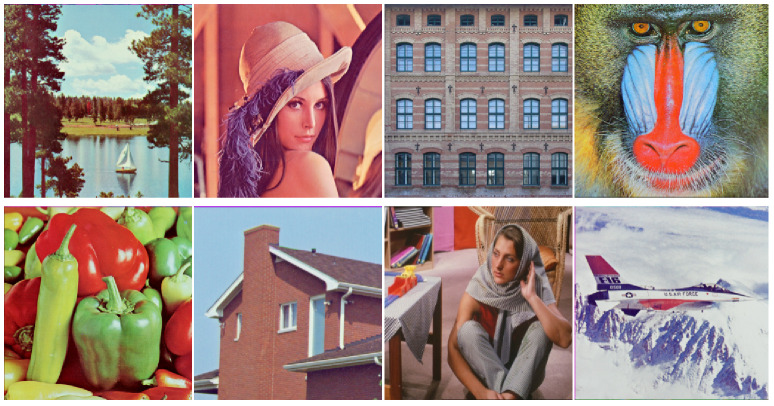
The eight benchmark color images.

In this section, we test the proposed TRSM-TC against other completion algorithms on eight benchmark color images shown in Figure 4. In recent years, reshaping low-order tensors into high-order tensors and then selecting an appropriate high-order form is a commonly used strategy to improve the performance of the TT/TR-based methods on visual-data completion [41,43]. To evaluate the performances of different methods in high-order forms and investigate in which forms they perform the best, we further reshaped the color images of size (256×256×3) (3D) to (16×16×16×16×3) (5D), (4×4×4×4×4×4×4×4×3) (9D) and visual data tensorization (VDT) (9D). The VDT tensorization has been introduced in [55]. The idea is simple, if the size of a RGB image is u×v×3 and $u=u_{1}\times u_{2}\times \cdots \times u_{l}$ and $v=v_{1}\times v_{2}\times \cdots \times v_{l}$ are satisfied, then the image can be tensorized to a (l+1) dimension tensor of size $u_{1}v_{1}\times u_{2}v_{2}\times \cdots \times u_{l}v_{l}\times 3$. In this experiment, we firstly reshape the three-way color image to a seventeen-way tensor of size 2×2×⋯×2×3 and permute the tensor according to order {1,9,2,10,3,11,4,12,5,13,6,14,7,15,8,16,17}. Then, we reshape the tensor to a nine-way tensor of size (4×4×4×4×4×4×4×4×3).

In this experiment, we set the random missing rate as 0.8 and 0.9 and evaluated the recovery performance using RSE, which is defined as

(21) $$\mathrm{RSE}={∥X-T∥}_{F}/{∥T∥}_{F},$$

where X is the ground-truth tensor, and T is the tensor recovered by the tensor completion algorithm. Smaller RSE means better recovery results. In addition to RSE, we also adopt a structural similarity index measure (SSIM) [56] to assess the performance of image recovery. A higher SSIM value indicates better recovery performance. For TR-related algorithms, we set the TR-ranks as the same value (i.e., $R_{1}=R_{2}=\cdots =R_{d}$), and we vary the TR-ranks to obtain the best results. The parameters of other algorithms are tuned to achieve the best performance.

Table 1 lists the averaged performances of the eight color images over different algorithms under different sampling rates and tensor shapes. It can be seen from Table 1 that the proposed method and other TR-based algorithms (i.e., TRLRF, TR-WOPT) obtain significantly lower RSE in 5D. These experiment results suggest that an appropriate high-order tensorization scheme does help improve the performances of TR-based methods. For other compared methods, we can observe that they achieve worse performances after reshaping into high-order forms. Compared with other methods, the proposed TRSM-TC obtains the best recovery performance in the 5D form. In the following experiments, we keep the 5D tensorization scheme for the TR-based methods and adopt the original data structure for the other methods. In Table 2, we compare the recovery SSIM between different methods. It is evident that the proposed method consistently achieves the highest SSIM across various missing rates, thereby demonstrating its effectiveness in recovering color images.

**Figure 5:**
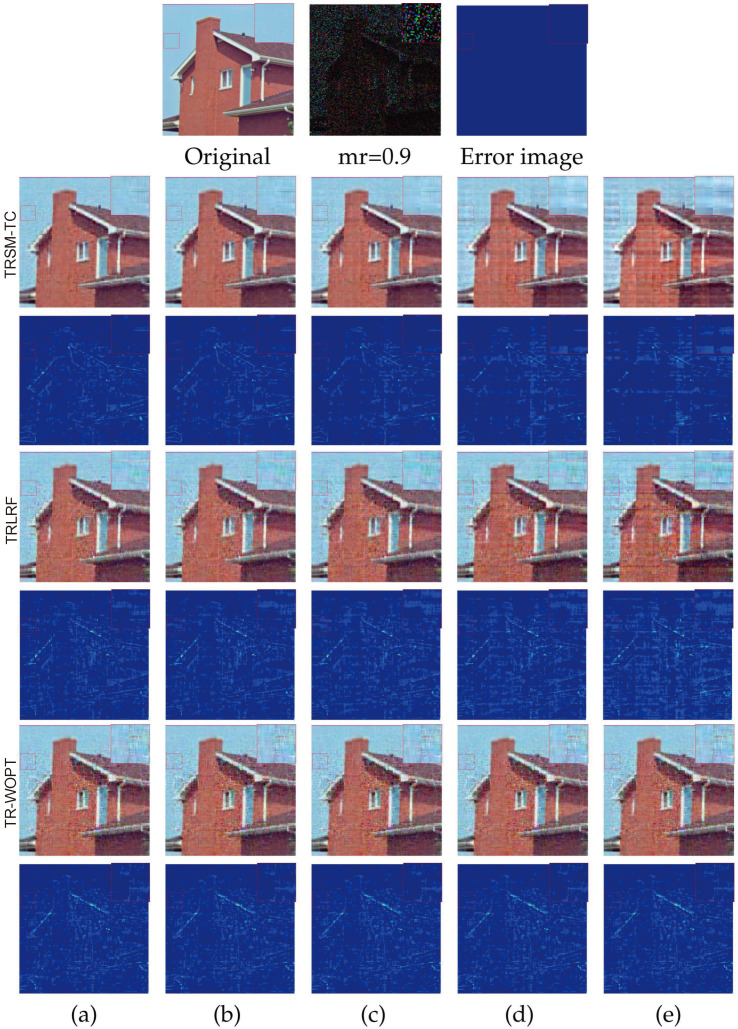
Visual completion result of the house image for a missing rate of 0.9 with 5D tensorization and TR-ranks 12. Top row from left to right: the original image, its 0.9 missing case, and the ground truth error image. The second and third rows show the images recovered by TRSM-TC and their corresponding error images, respectively; the fourth and fifth rows show the images recovered by TRLRF and their corresponding error images, respectively; the sixth and seventh rows show the images recovered by TR-WOPT and their corresponding error images, respectively. (**a**) The fully recovered images. (**b**) The images recovered by the first 230,000 Kronecker bases. (**c**) The images recovered by the first 220,000 Kronecker bases. (**d**) The images recovered by the first 210,000 Kronecker bases. (**e**) The images recovered by the first 200,000 Kronecker bases. In each figure, we zoom in on the small red box located on the left and display the result in the upper right area.

In Figure 5, we visualize the house image recovered under 90 missing rates by a portion of the Kronecker bases constructed by the learned TR-cores from different TR-based completion methods. Besides showing the fully recovered tensor constructed by the TR-related algorithms, the recovered images constructed from the first 200,000, 210,000, 220,000, 230,000 largest Kronecker bases (ranked based on the Frobenius norm of the Kronecker basis) are also demonstrated. As we can see, when adding more Kronecker bases, the recovery results of all the methods improve. However, the improvements in TR-WOPT and TRLRF are relatively small. Moreover, TRSM-TC consistently achieves better recovery performance compared with TR-WOPT and TRLRF under the same number of Kronecker bases. These results indicate that the proposed TRSM-TC is encouraged to find more meaningful and expressive TR Kronecker bases.

### 6.2. Noisy Color Images Completion

In practice, there is additive noise in the image data. Therefore, in this section, we tend to test the robustness of the proposed method to the noise. By selecting eight benchmark color images described in the last section as the ground truth images, we add Gaussian noise with different intensities to them according to signal–noise ratios (SNR) of 21, 26, and 31. Then, we set the random missing rate as 0.8 and 0.9 and evaluate the recovery performance of different methods using RSE and SSIM.

In Table 3, we demonstrate the averaging recovery performances of different methods over eight color images under different missing rates and noise. From the result, we can see that the recovery performances of all the methods become worse than their noise-free cases. However, we can see that the proposed TRSM-TC still outperforms the other testing methods in the noisy cases in terms of both RSE and SSIM.

### 6.3. Multispectral Images Completion

In this section, we use the Columbia Multispectral Image Database (CAVE) [57], which contains multispectral images of 32 real-world scenes, each with a spatial resolution of 512×512 and 31 bands (varying from 400 nm to 700 nm in 10 nm steps). Each image is resized to 256×256 for all spectral bands and rescaled to [0, 1]. For the TR-related methods, the multispectral images of size (256×256×31) (3D) are directly reshaped to (16×16×16×16×31) (5D).

In this experiment, we considered the high missing rates of 0.95 and 0.98. RSE, SSIM, and *erreur relative globale adiensionnelle de synthese* (ERGAS [58]) were employed for performance evaluation, and ERGAS is defined as

(22) $$\mathrm{ERGAS}=100\sqrt{\frac{1}{s}\sum_{i=1}^{s}\frac{\mathrm{MSE}(X_{::i},T_{::i})}{\mathrm{MEAN}{\left(T_{::i}\right)}^{2}}},$$

where *s* is equal to the spectral dimension of the tensor. $X_{::i}$ and $T_{::i}$ are the *i*-th frontal slice of ground-truth tensor X and recovery tensor T. Moreover, MSE(·) is the mean square error operation of two matrices and MEAN(·) is the mean value operation of the matrix. For ERGAS, good completion results correspond to smaller values.

The averaged performances of 32 multispectral images under different sampling rates are summarized in Table 4. As shown in Table 4, TRSM-TC performs the best with respect to all the evaluation metrics. Moreover, it should be noted that it is challenging for image completion algorithms to recover the images with a 0.98 missing rate. While most of the algorithms fail to achieve satisfactory completion performances, the proposed method still achieves a high performance.

**Figure 6:**
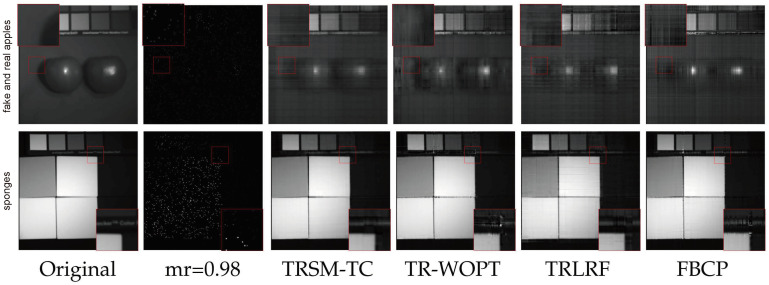
Visual completion result of the 31st band of fake and real apples and the 29th band of sponges of the 0.98 missing rate. The first and second rows represent the recovery results of fake and real apples and sponges, respectively, and from left to right are the original images, the 0.98 missing rate case of the images, and the images recovered by algorithms TRSM-TC, TR-WOPT, TRLRF, and FBCP, respectively. In each figure, we zoom in on the small red box and display the result in the larger red box.

In order to visually compare the performances of the competing methods with a 0.98 missing rate, we show in Figure 6 the 31st band of fake and real apples and the 29th band of sponges. We only demonstrate the results of TRSM-TC, TR-WOPT, TRLRF, and FBCP because other methods fail in this case. From the figures, the superiority of the proposed method can be observed.

### 6.4. Videos Completion

In the video completion experiment, six videos of size (112×160×3×32) (4D) were chosen from the video trace library [59]. For the TR-related methods, we reshaped them to (8×14×16×10×3×32) (6D).

**Figure 7:**
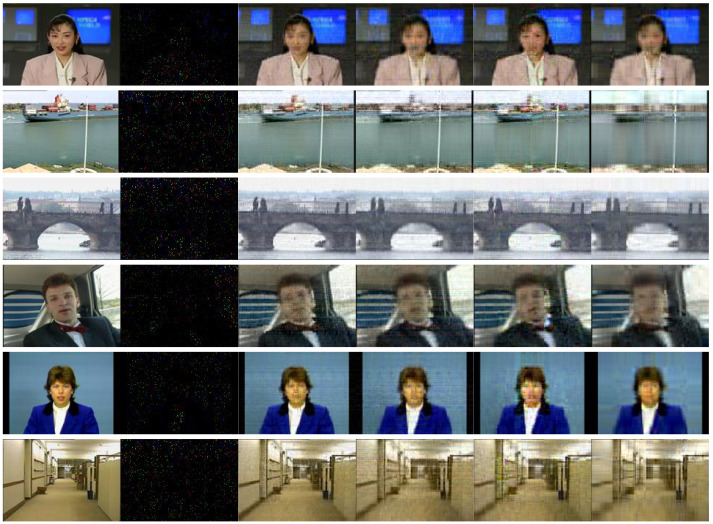
Visual completion results of 6 videos under a 0.98 missing rate. The columns from left to right: the original videos, the 0.98 missing cases and the videos recovered by algorithms TRSM-TC, TRLRF, TR-WOPT, and FBCP, respectively.

To further demonstrate the superiority of the proposed method in recovering data with a high missing rate, we consider that only 2% of the video data are observed, and the average completion results are shown in Table 5. From the table, we can see that TRSM-TC achieves the lowest RSE and ERGAS and the highest SSIM compared with other methods. Moreover, the visual completion results of TRSM-TC, TRLRF, TR-WOPT, and FBCP are demonstrated in Figure 7. It can be seen from the results that the proposed method outperforms all the other algorithms. In particular, the proposed method can recover the finer-grained textures and coarser-grained structures of the videos while the other methods can not.

**Figure 8:**
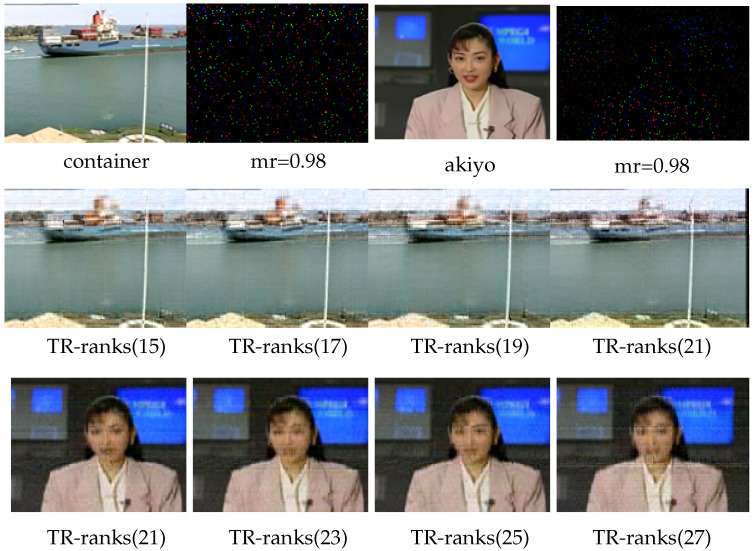
Visual completion result of the 3rd frame of the container and the 24th frame of akiyo using TRSM-TC. The top row shows the original images and its 0.98 missing cases for container and akiyo. The second and third rows show the recovered results of container and akiyo under different TR-ranks.

Furthermore, since the proposed method includes an explicit low-rank constraint on the TR-cores, the resulting TR decomposition tends to be low-rank, even when larger initial TR-ranks are set. In the following experiment, we aim to test the rank robustness of the proposed algorithm. As we can see in Figure 8, the recovery results of container and akiyo under different choices of TR-ranks are shown. The best completion results for container and akiyo are obtained when TR-ranks are set as 15 and 21, respectively. After that, with the increase in TR-ranks, the recovery performances are bound to decline due to the overfitting. However, the results indicate that the performance remains relatively stable. Specifically, in the case of the container’s recovery, even when the TR-ranks increase to 21—which is 6 higher than the best ranks—the overall structure of the image is still preserved. These findings confirm the rank robustness of the proposed method.

### 6.5. Parameters Analysis

**Figure 9:**
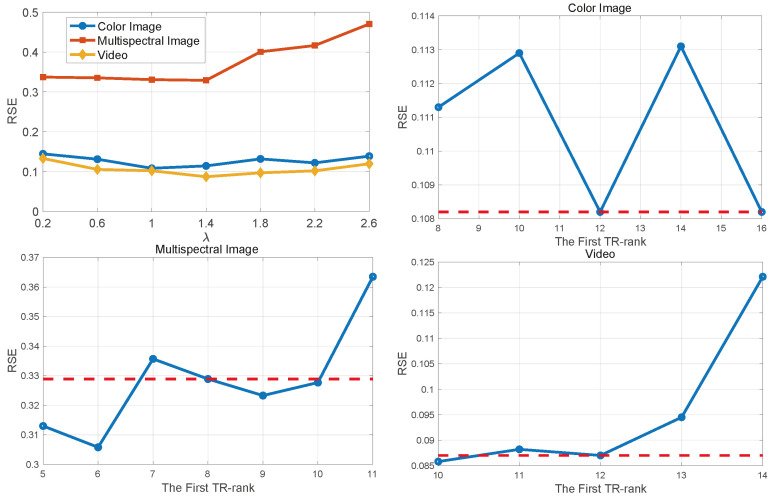
Ablation studies examining the effects of λ and the first TR-rank. In figures focusing on the first TR-rank, the horizontal dashed line represents results achieved when the first TR-rank is set equal to the other ranks.

In this section, our objective is to investigate the effects of varying λ, which is the hyperparameter that controls the MCP penalty level, and $R_{1}$, the first TR-rank, on the recovery performance of our proposed method. We conduct our study using one image each from color, multispectral, and video datasets with missing rates of 0.9 for the color image and 0.98 for both the multispectral image and video. Furthermore, we set the TR-ranks to 12 for the color image and video, and 8 for the multispectral image. As we can see from the upper left figure in Figure 9, the RSE for both datasets initially decreases and then increases as λ surpasses a certain threshold. This suggests that both too small and too large values of λ can negatively impact the method’s performance. The underlying reason is that λ governs the regularization intensity of the penalty term in the objective function. From the results, it is recommended to choose a value for λ within [0.6,1.4]. To evaluate the effect of varying $R_{1}$, we keep all the other TR-ranks fixed and only adjust the value of the first TR-rank. The results presented in Figure 9 indicate that tuning the first TR-rank has a noticeable impact on performance across various datasets, with the most pronounced improvement observed in the multispectral image case, and this is because most of the redundancy of the multispectral image exists in the spectral mode [60], which is controlled by the first TR-rank. These findings imply that tuning the first TR-rank independently could be beneficial in practical applications, particularly when significant low-rankness is present in either the first or the last mode of the tensor.

## 7. Conclusions

In this paper, we propose a novel tensor sparsity measurement based on TR decomposition, termed TRSM. The proposed TRSM has a unified interpretation with a matrix sparsity measurement by taking the Kronecker basis as the fundamental representation component. By incorporating TRSM in the tensor completion problem as an optimization objective and solving it with the modified ADMM, we conducted extensive image and video data completion experiments to demonstrate the effectiveness of the proposed TRSM.

In our future research, we will employ TRSM to TRPCA and other tensor analysis applications to improve their performance.

## Figures and Tables

**Table 1:** The average performance comparison of 7 competing TC methods with different missing rates on 8 color images. All the methods’ best performances over different tensorization schemes are highlighted in bold.

Methods	mr = 0.8				mr = 0.9			
	3D	5D	9D	VDT	3D	5D	9D	VDT
CP-WOPT	**0.1402**	0.1607	0.2335	0.2403	**0.2038**	0.2129	0.2663	0.2849
FBCP	**0.1343 **	0.1551	0.2343	0.2532	**0.1799 **	0.1984	0.2716	0.2812
HaLRTC	**0.1327 **	0.2801	0.3276	0.3291	**0.1889**	0.3309	0.3565	0.3581
Tmac	**0.1242 **	0.1373	0.1326	0.1463	**0.1785 **	0.1853	0.1964	0.2002
TRLRF	0.1279	**0.1148 **	0.1299	0.1349	0.1972	**0.1610 **	0.1646	0.1982
TR-WOPT	0.1396	**0.1044 **	0.1143	0.1278	0.2092	0.1434	**0.1433 **	0.1695
TRSM-TC	0.1343	**0.0925 **	0.1320	0.1340	0.2179	**0.1283 **	0.1734	0.1716

**Table 2:** The averaged recovery SSIM between 7 competing TC methods with different missing rates on 8 color images. The best results are highlighted in bold.

MR	CP-WOPT	FBCP	HaLRTC	Tmac	TRLRF	TR-WOPT	TRSM-TC
0.8	0.5589	0.6341	0.6726	0.6348	0.6814	0.6981	**0.7627**
0.9	0.4003	0.4469	0.4946	0.4492	0.493	0.551	**0.6174 **

**Table 3:** The average performance comparison of 7 competing TC methods with different missing rates and SNR on 8 color images. The upper and the lower tables show the recovery performances under missing rates 0.8 and 0.9, respectively. The best RSE and SSIM are highlighted in bold.

SNR	CP-WOPT		FBCP		HaLRTC		Tmac		TRLRF		TR-WOPT		TRSM-TC	
	RSE	SSIM	RSE	SSIM	RSE	SSIM	RSE	SSIM	RSE	SSIM	RSE	SSIM	RSE	SSIM
31	0.143	0.546	0.137	0.622	0.136	0.658	0.128	0.621	0.117	0.674	0.108	0.691	**0.098**	**0.740**
26	0.147	0.543	0.138	0.597	0.141	0.632	0.136	0.590	0.121	0.650	0.112	0.667	**0.103**	**0.719**
21	0.156	0.495	0.147	0.540	0.153	0.580	0.158	0.508	0.133	0.602	0.123	0.618	**0.114**	**0.657**
SNR	CP-WOPT		FBCP		HaLRTC		Tmac		TRLRF		TR-WOPT		TRSM-TC	
	RSE	SSIM	RSE	SSIM	RSE	SSIM	RSE	SSIM	RSE	SSIM	RSE	SSIM	RSE	SSIM
31	0.208	0.402	0.184	0.440	0.192	0.487	0.182	0.445	0.163	0.529	0.145	0.553	**0.129**	**0.604**
26	0.210	0.393	0.186	0.428	0.195	0.475	0.187	0.426	0.164	0.525	0.148	0.535	**0.133**	**0.584**
21	0.221	0.367	0.191	0.397	0.205	0.443	0.205	0.379	0.172	0.482	0.156	0.498	**0.143**	**0.540**

**Table 4:** The average performance comparison of 7 competing TC methods with different missing rates on 32 MSI images. The best results achieved over different recovery metrics are highlighted in bold.

MR		CP-WOPT	FBCP	HaLRTC	Tmac	TRLRF	TR-WOPT	TRSM-TC
0.95	RSE	0.1835	0.1352	0.3343	0.1764	0.1381	0.1344	**0.1162**
	ERGAS	164.1529	123.0992	309.2216	163.4394	128.0540	123.4724	**107.6782**
	SSIM	0.8506	0.8716	0.7672	0.8251	0.853	0.8805	**0.896**
0.98	RSE	0.6466	0.2374	0.6314	0.3901	0.2696	0.2308	**0.1980**
	ERGAS	522.4653	216.4430	591.0829	354.5717	249.1188	210.0180	**182.5890**
	SSIM	0.4372	0.7461	0.4469	0.6186	0.6653	0.7700	**0.7754**

**Table 5:** The average video completion performances of 7 competing TC methods with a missing rate of 0.98. The best results achieved over different recovery metrics are highlighted in bold.

	CP-WOPT	FBCP	HaLRTC	Tmac	TRLRF	TR-WOPT	TRSM-TC
RSE	0.1775	0.1165	0.4204	0.2316	0.1067	0.1082	**0.0858 **
ERGAS	218.6831	143.0337	515.8984	286.2220	131.8164	133.7670	**107.7512 **
SSIM	0.5941	0.7413	0.4073	0.5392	0.7493	0.7432	**0.8079 **

## Data Availability

No new data were created or analyzed in this study. Data sharing is not applicable to this article.
